# Agonistic β-Klotho antibody mimics fibroblast growth factor 21 (FGF21) functions

**DOI:** 10.1074/jbc.RA118.004343

**Published:** 2018-08-01

**Authors:** Xiaoshan Min, Jennifer Weiszmann, Sheree Johnstone, Wei Wang, Xinchao Yu, William Romanow, Stephen Thibault, Yang Li, Zhulun Wang

**Affiliations:** From the ‡ Department of Therapeutic Discovery and; § Department of Cardiometabolic Disorders, Amgen Discovery Research, Amgen Inc., South San Francisco, California 94080

**Keywords:** crystallography, antibody, fibroblast growth factor (FGF), metabolism, electron microscopy (EM), β-Klotho, agonist, antibody, binding

## Abstract

Fibroblast growth factor 21 (FGF21), an endocrine hormone in the FGF family, plays a critical role in regulating metabolic homeostasis and has emerged as a therapeutic target for metabolic diseases, including Type 2 diabetes mellitus. FGF21 functions through a receptor complex that consists of an FGF receptor (FGFR) and a co-receptor β-Klotho. Here, we identify and biochemically and structurally characterize 39F7, a high-affinity agonistic monoclonal antibody (mAb) against β-Klotho that mimics FGF21 function. The co-crystal structure of β-Klotho KL1 domain in complex with 39F7 Fab revealed that the recognition of 39F7 is centered on Trp-295 of β-Klotho in a FGF21 noncompetitive manner. KL1 adopts a (β/α)_8_ TIM barrel fold which resembles that of β-glycosylceramidase, but lacks molecular features for enzymatic activity, suggesting that KL1 functions as a scaffold protein instead. *In vitro* characterization demonstrated that, although 39F7 does not compete with FGF21, it is specific for β-Klotho/FGFR1c activation. Furthermore, the agonistic activity of 39F7 required the full IgG molecule to be bivalent, suggesting that 39F7 functions by promoting receptor/co-receptor dimerization. Supported by negative stain EM analysis of full-length β-Klotho, we propose a molecular model wherein the agonistic antibody 39F7 acts in a β-Klotho– and FGFR1c-dependent manner, mimicking FGF21 activity. More importantly, 39F7 offers promising therapeutic potential in the axis of FGF21 signaling as an antibody therapy alternative to FGF21 analogs for treatment of metabolic diseases.

## Introduction

Fibroblast growth factors (FGFs) are a group of secreted molecules that serve a wide range of functions throughout the human development. There are 18 mammalian FGFs that can be divided into six subfamilies (1–3). Five of the subfamilies are considered paracrine factors that, because of their high affinity toward the extracellular matrix (ECM) ^4^ component heparan sulfate (HS), are retained in the ECM and function locally. The sixth subfamily members, including FGF19, FGF21, and FGF23, are distinct from the rest of the FGFs in that they have reduced affinity toward HS and, therefore, can escape from the ECM and function as hormones. Hence, members of the sixth subfamily are also called endocrine FGFs. FGF receptors (FGFRs) are encoded by four genes (*FGFR1, FGFR2, FGFR3,* and *FGFR4*) and are single-pass transmembrane receptors of the tyrosine kinase family (RTKs). Alternative splicing in the extracellular domain of *FGFR1–3,* but not *FGFR4,* results in “b” and “c” isoforms. Paracrine FGFs utilize HS as a cofactor for high-affinity interaction with FGFRs and to activate receptor signaling. For endocrine FGFs (eFGF), however, instead of HS, two transmembrane proteins (α-Klotho and β-Klotho) have been shown as obligate co-receptors for signaling. Whereas FGF19 and FGF21 require β-Klotho to regulate glucose, lipid, and energy metabolism, FGF23 functions through α-Klotho to maintain phosphate homeostasis (4).

The α-Klotho protein was initially identified from knockout mice that exhibited short life span and phenotypes resembling human premature syndromes (5). Later, it was found that α-Klotho can form binary complexes with FGFR1c, FGFR3c, and FGFR4 (6). The β-Klotho protein was cloned subsequently based on sequence identity with α-Klotho, and the knockout mutant mice carry phenotypes that are reminiscent of FGFR4 or FGF19 knockouts with respect to bile acid regulation (7). Whereas α-Klotho is predominantly expressed in the kidney and brain, β-Klotho is more restricted to the liver and the fat tissues (8). It is believed that these two co-receptors, α-Klotho and β-Klotho, serve primarily as docking sites for the endocrine FGFs to facilitate their interactions with FGFRs and subsequent signaling (9, 10). Both α- and β-Klotho are single-pass membrane proteins with very short intracellular domains. The extracellular domain of the Klotho protein contains two tandem repeats, named KL1 and KL2. The KL1 and KL2 domains share low sequence identity with the glycosidase family, which includes lactase-phlorizin hydrolase and β-glucosidase. Because of the divergence among the active site residues, it is generally believed that the KL1 and KL2 domains in the Klotho family do not possess enzymatic activities. However, the enzymatic activity of α-Klotho remains controversial as a few reports suggested that α-Klotho protein retains enzymatic activity despite catalytic residue mutations (11–13).

Multiple structures of FGFs, FGFRs, and complexes between FGFs and FGFRs have been determined over the years (14, 15). These structural studies have revealed detailed interactions between paracrine FGFs and FGFRs and thus provided molecular insights for paracrine FGF functions. For endocrine FGFs, only very recently, the crystal structures of the complex of a FGF21 C-terminal peptide with β-Klotho and the complex of FGF23 with α-Klotho and FGFR1c have been determined (16, 17). These structures begin to shed light on the interaction of endocrine FGFs with the co-receptor protein and receptor.

In the endocrine FGF19 subfamily, of particular interest is FGF21, which is produced mainly in the liver and signals through FGFR1c and β-Klotho. Numerous pharmacological studies have shown that FGF21 regulates glucose and lipid metabolism and demonstrates positive effects in the management of diabetes (18, 19). A number of FGF21 analogs have entered clinical development (20, 21). The future of FGF21 therapy is to be determined because of its versatile role in the complex networks and potential undesirable side effects.

In a search for an alternative FGF21 therapy, we previously described an agonistic monoclonal antibody (mAb) that binds to β-Klotho and demonstrated FGF21-like metabolic effect in monkey (22). Here, we report the identification, biochemical characterization, and mechanistic study of 39F7, another β-Klotho–binding agonistic mAb. We determined the crystal structure of β-Klotho KL1 domain in complex with 39F7 Fab. Negative-stain EM was employed to study the full-length β-Klotho alone as well as in the presence of 39F7 Fab or endogenous ligand FGF21. Based on these data, we propose a signaling model for mAb 39F7 through a β-Klotho– and FGFR1c-dependent manner, resembling FGF21 action. Thus, 39F7 might offer promising therapeutic potential for treating diabetes and obesity in the FGF21 signal pathway.

## Results

### Identification of a high-affinity agonistic antibody against β-Klotho

**Figure 1.**
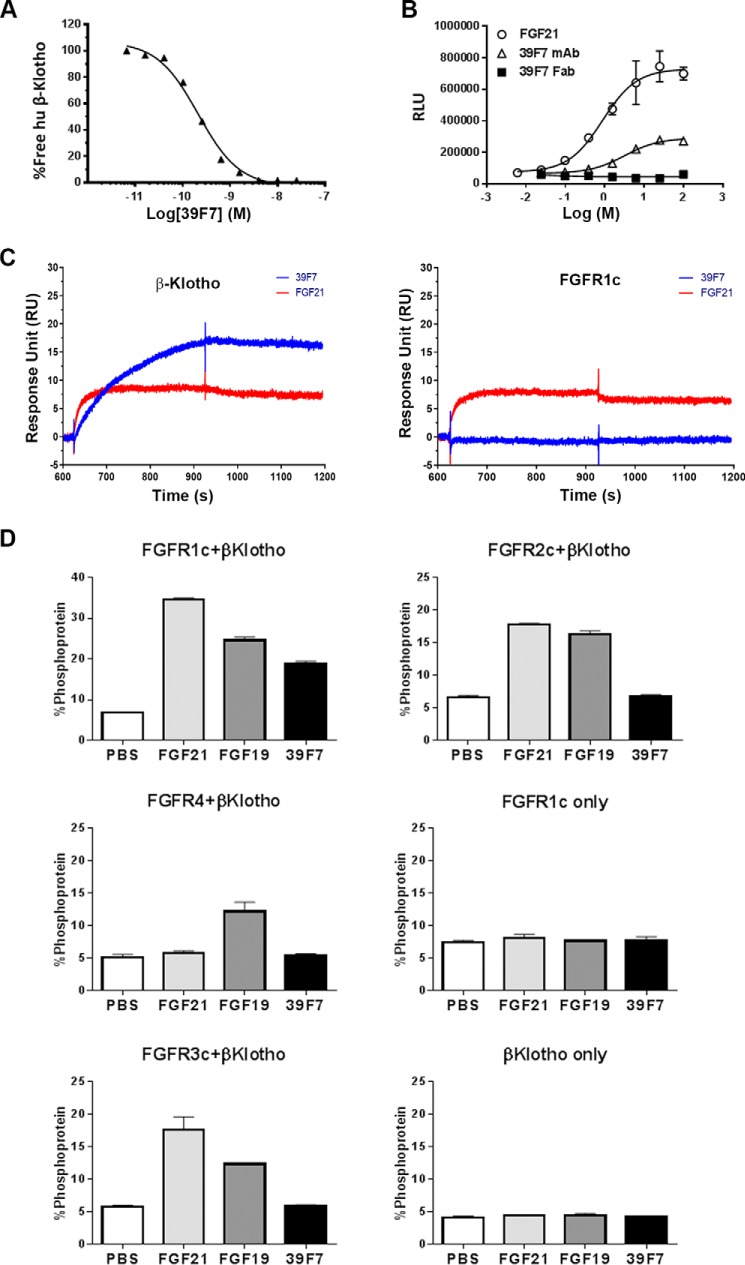
**Biochemical characterization of 39F7.** *A*, binding affinity of 39F7 to human β-Klotho by solution equilibrium assay on Biacore. Human β-Klotho at 10 nm was premixed with 39F7, and the binding of free β-Klotho was detected by injecting over immobilized 39F7 surface on Biacore T200. Shown here, binding of 10 nm β-Klotho alone as 100%, the relative binding of β-Klotho in the mix with 39F7 was plotted *versus* antibody concentration. *B*, FGF21, 39F7, or 39F7 Fab induces luciferase activity in an FGF21-responsive reporter cell line. Luciferase activity is measured in relative luminescence units (*RLU*). *C*, binding sensorgram of 39F7 to human β-Klotho and FGFR1c on Biacore. Human β-Klotho and FGFR1c were captured at an approximate density of 40 RU and 60 RU on an anti-His antibody surface. 39F7 (*blue*) and FGF21 (*red*) at 100 nm and 500 nm were injected over captured β-Klotho and FGFR1c. *D*, 39F7 requires FGFR1c and β-Klotho to induce signaling as measured by ERK phosphorylation in L6 cells. L6 cells were selected for this characterization, as they do not express any endogenous FGF receptors. Data are expressed as percent ERK protein that is phosphorylated, as measured by Meso Scale Discovery (MSD) assay.

We previously described an immunization campaign in XenoMouse that resulted in the identification of “mimAb1” that specifically activates the β-Klotho/FGFR1c receptor complex (22). From the same campaign, we identified another high-affinity antibody, 39F7, with unique properties that are distinctive from mimAb1. 39F7 binds human β-Klotho with a *K_D_* value of 86 picomolar as determined by surface plasmon resonance (SPR) analysis (Fig. 1*A*) and activates FGF receptor activity in a FGF21-responsive reporter cell assay (Fig. 1*B*). Interestingly, such agonistic activity requires the presence of both arms of the antibody because the monovalent Fab of 39F7 is unable to activate receptor signaling in the reporter cell assay (Fig. 1*B*).

Although the immunization campaign was performed with a complex of β-Klotho and FGFR1c as an immunogen, 39F7 did not show significant affinity toward a recombinant FGFR1c extracellular domain protein in a SPR-binding study (Fig. 1*C*). Even though 39F7 does not directly bind to FGFR1c with measurable affinity, it does, however, show specificity toward the receptor in a functional assay. Human FGFR1c, 2c, 3c, and 4 were separately co-transfected with human β-Klotho into rat L6 cells, and the pERK levels were measured after treatment with FGF19, FGF21, or 39F7. Although both FGF19 and FGF21 activated FGFR1c, 2c, and 3c in the presence of β-Klotho, and FGF19 additionally activated FGFR4 as reported previously (23), 39F7 activated β-Klotho/FGFR1c only (Fig. 1*D*). Such an activation also required the presence of both β-Klotho and FGFR1c because L6 transfected with either receptor alone was unresponsive to treatments (Fig. 1*D*).

**Figure 2.**
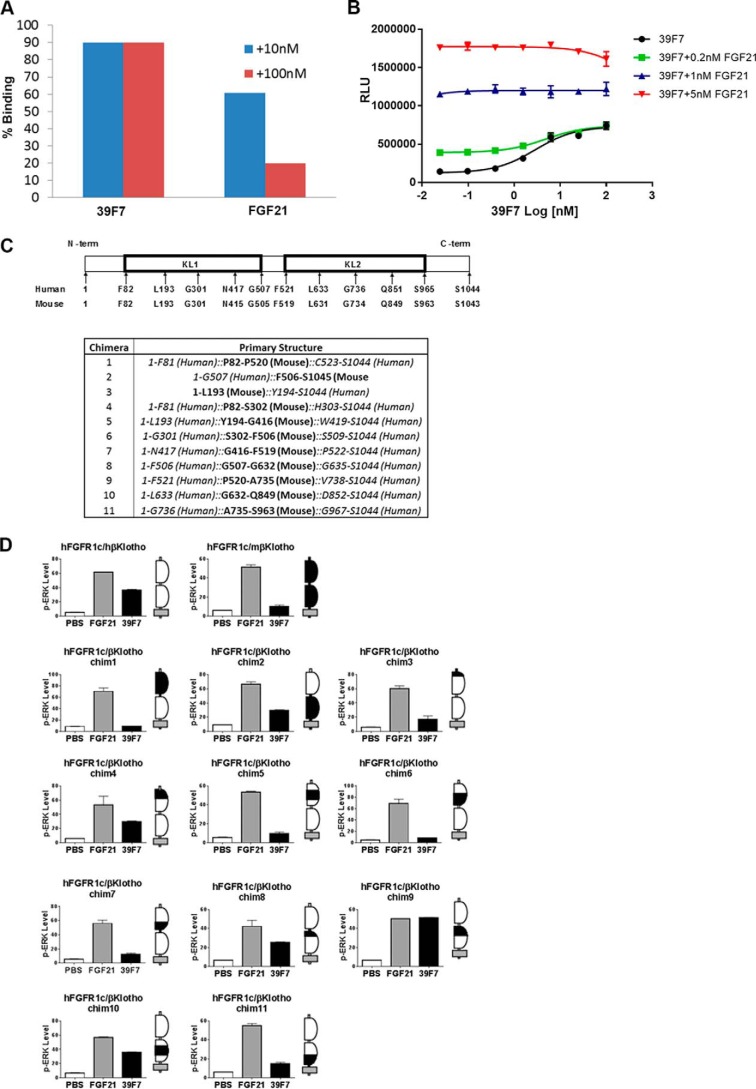
**39F7 binds to β-Klotho KL1 domain and does not compete with FGF21 binding to the co-receptor.** *A*, competition of 39F7 with FGF21 on the binding to β-Klotho. Human β-Klotho at 10 nm was premixed with 39F7 or FGF21, and the binding of free β-Klotho was detected by injecting over immobilized FGF21 on Biacore T200. Using binding of 10 nm β-Klotho alone as 100%, the relative binding of β-Klotho premixed with 39F7 or FGF21 at different concentrations, 10 nm (*blue*) or 100 nm (*red*), was plotted. The data shown are representative of multiple repeats. *B*, the 39F7-induced luciferase reporter activity was tested in the absence or presence of different concentrations of FGF21. *C* and *D*, a series of human–mouse β-Klotho chimeras was designed (*C*) and co-transfected into L6 cells with human FGFR1c, and signaling was measured by ERK phosphorylation (*D*). The β-Klotho chimeras are represented by schematics next to the pERK histograms, with murine sequence indicated as *black* and human sequence represented as *white*. The plasma membrane is indicated in *gray*.

To provide molecular insights into the 39F7 recognition of β-Klotho, we first sought to understand whether 39F7 binding competes with an endogenous ligand. This was first assessed using SPR method. β-Klotho alone, or β-Klotho preincubated with 10 nm or 100 nm of 39F7, or FGF21 was injected over a Biacore sensor chip immobilized with FGF21. As shown in Fig. 2*A*, although preincubation of β-Klotho with 39F7 did not affect β-Klotho's ability to bind to the FGF21 chip, preincubation of β-Klotho with soluble FGF21 competed with β-Klotho binding to FGF21 on the chip surface. This result suggests that 39F7 and FGF21 bind to different binding sites on the co-receptor β-Klotho. The potential interaction between FGF21 and 39F7 was also studied in a cell-based setting. In the FGF21-responsive reporter cells, 39F7 dose responses were performed in the presence of different amounts of FGF21. Because 39F7 is a partial agonist, as shown in Fig. 1*B*, if the 39F7-binding site overlaps with the FGF21 site, 39F7 would compete with FGF21 binding and result in an inhibition curve at higher FGF21 concentrations. As shown in Fig. 2*B*, 39F7 does not compete with FGF21 at all doses tested, suggesting that 39F7 is noncompetitive to endogenous ligand binding to β-Klotho.

To further understand the binding site of 39F7 on β-Klotho, we took advantage of the high specificity of 39F7 toward human β-Klotho. As shown in Fig. 2*D*, 39F7 activated signaling in L6 cells co-transfected with human β-Klotho and FGFR1c, but not mouse β-Klotho and FGFR1c. A series of chimeric β-Klotho proteins between human and mouse sequences were constructed (Fig. 2*C*) and tested in the presence of FGF21 and 39F7. FGF21 activated all the chimeric co-receptors demonstrating that all the chimeras were expressed and functional (Fig. 2*D*), however, the lack of activities from 39F7 on these chimeric proteins would then suggest the regions mutated to mouse sequences might be important for 39F7 interaction. Analysis of these chimeric mutant receptors showed that KL1 is the predominant binding domain for 39F7 as substitution with mouse KL1 completely abolished activity (chim 1) whereas substitution with mouse KL2 had little impact (chim 2). This is further confirmed by the loss of activity with additional chimeras in the KL1 region (chims 3 and 5–7), whereas chimera in the extreme N-terminal region of KL1 (chim 4) and additional chimeras in the KL2 region (chims 8 and 9) were tolerated (Fig. 2*D*). These *in vitro* characterizations together suggest that 39F7 is a β-Klotho/FGFR1c–specific agonist that does not compete with endogenous ligand, and predominantly binds to the KL1 region of β-Klotho.

### Crystal structure of KL1–39F7 Fab complex

**Figure 3.**
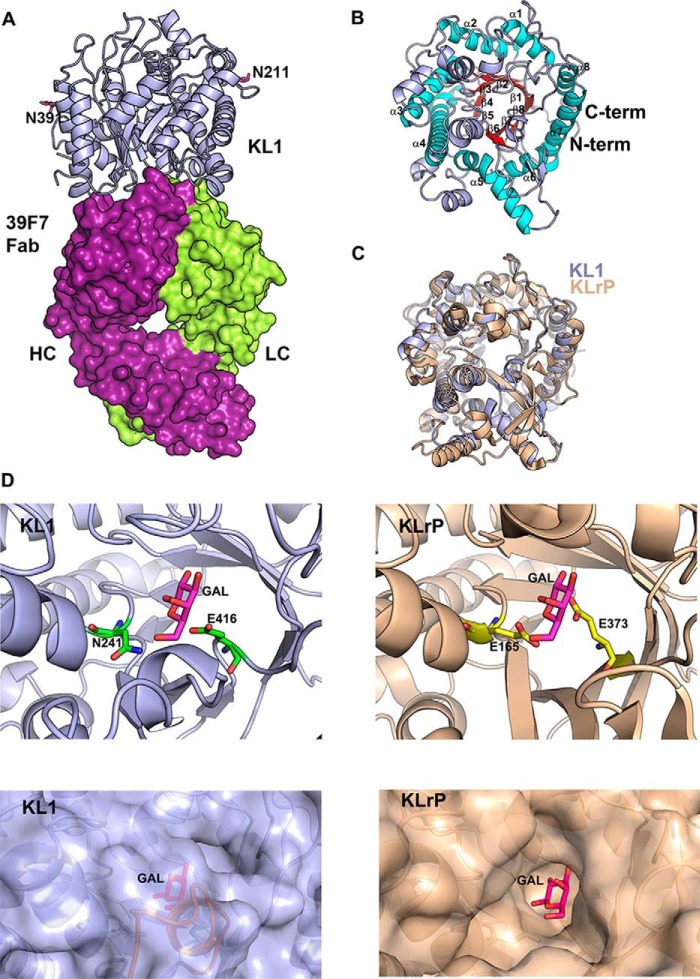
**Structure of 39F7-KL1 complex.** *A*, overall structure of 39F7 Fab/KL1 complex. KL1 domain is shown in cartoon representation and colored in *light blue*. 39F7 Fab is shown in surface representation and colored in *purple* and *green* for heavy chain and light chain, respectively. *B*, structure of KL1. KL1 is shown in cartoon representation and is colored by secondary structure: *red* for β-strand and *cyan* for α-helix in the TIM barrel fold and *light blue* for loop and additional helices not belonging to the TIM barrel fold. *C*, overlay of KL1 domain with KLrP protein (PDB ID: 2E9L). KL1 domain is colored in *blue* and KLrP protein is colored in *wheat. D*, comparison of the active site of KLrP and KL1 domain. *Top panels*: the KLrP protein is shown in *wheat* cartoon and the active site residues are shown as *yellow sticks*. Galactose is shown as *magenta sticks*. KL1 domain protein is shown as *light blue* cartoon and the corresponding active site residues are shown as *green sticks*. A modeled glucose molecule is shown in the KL1 active site in *magenta sticks. Bottom panels*: KLrP protein is shown as *wheat* surface and the KL1 domain protein is shown as *light blue* surface. Galactose molecule in KLrP and the modeled galactose molecule in KL1 are shown as *magenta sticks*. The peptide from 374 to 381 in KL1 is colored in *orange*.

Crystallization of the full-length extracellular domain of β-Klotho (residues 52–967) proved to be very challenging because of a heterogeneous glycosylation problem. We then generated the KL1 domain (residues 52–521) in insect cells at high expression level and purified it to homogeneity. We crystallized the KL1 domain in complex with 39F7 Fab and solved the structure by molecular replacement method using an internally produced model of KL1 as a search model (24). There are two KL1–39F7 Fab complexes in the asymmetric unit arranged in a head-to-head conformation (Fig. 3*A*). The two complexes are highly similar, with an r.m.s.d. of 0.226 Å. For each KL1 domain, the N-terminal residues from 52 to 77 and a loop between residues 119 and 124 are disordered. Because the KL1 protein was expressed in insect cells, there were six potential glycosylation sites, including N71, N120, N125, N211, N308, and N391. We observed partial glycan density for two of the sites, *i.e.* N211 and N391 (Fig. 3*A*).

The KL1 domain adopts a classical (β/α)_8_ TIM barrel fold with eight β-strands forming the inside of the barrel and eight helices forming the outside layer (Fig. 3*B*). We compared the structure of KL1 with that of KLrP (KL-related protein), a cytosolic neutral β-glycosylceramidase that shares 37% sequence identity with KL1 domain (24). Both KLrP and KL1 adopt typical TIM barrel folds with an r.m.s.d. of 0.8 Å, demonstrating a highly similar overall architecture (Fig. 3*C*), despite the low sequence similarity. In KLrP, two glutamate residues, Glu-165 and Glu-373 located in β-strands 4 and 7, respectively, play important roles for its catalytic activity. The first acidic residue, Glu-165 from β-strand 4, serves as an acid/base catalyst in glycosidase. The corresponding residue in KL1 is an asparagine Asn-241 that would be expected to abolish enzymatic activity. KLrP also possesses a channel extended from the active site to the solvent for substrate entry and product exit. In KL1, this channel is blocked by a small peptide loop encompassing residues 374–381 (Fig. 3*D*). Collectively, based on the structural features at the catalytic site, KL1 is not expected to be able to catalyze enzymatic reactions. This finding is consistent with that of the recently reported structure of full-length β-Klotho (17). When we compared the structure of KL1 domain in complex with 39F7 to the recently published structure of apoKL1 domain (PDB ID: 5VAK), the two structures are highly similar, with an r.m.s.d. of 0.24 Å (Fig. S1), suggesting that the binding of antibody 39F7 does not induce any significant conformational changes in β-Klotho.

**Figure 4.**
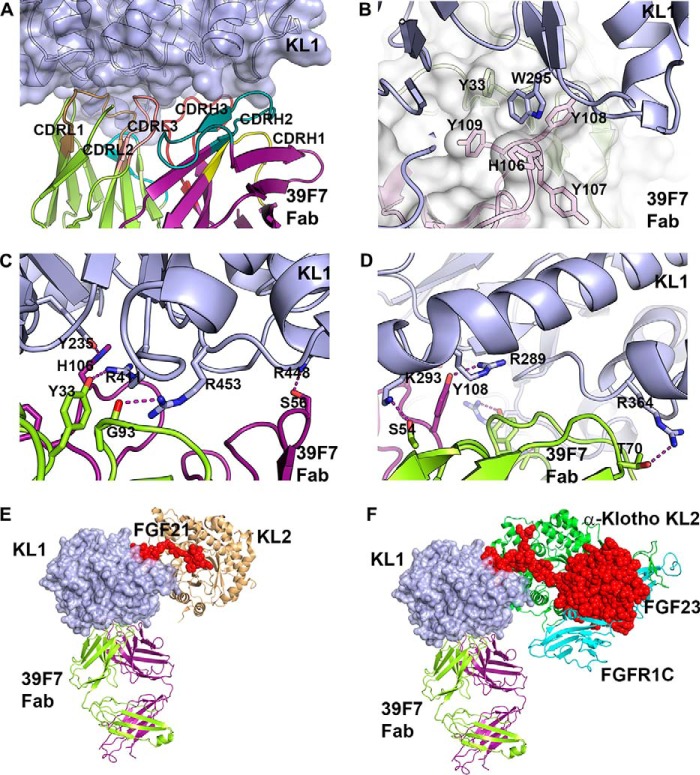
**Interactions between 39F7 Fab and KL1 domain.** *A*, the interface between KL1 and 39F7 Fab. KL1 protein is colored in *light blue*. 39F7 Fab is colored in *purple* for heavy chain and *green* for light chain. The six CDR loops are colored in the following order: CDRH1, *yellow*; CDRH2, *green*; CDRH3, *red*; CDRL1, *dark yellow*; CDRL2, *cyan*; CDRL3, *pink. B*, detailed hydrophobic interactions at the interface. *C* and *D*, H-bond interactions at the interface. KL1 domain is shown as *light blue* cartoon. 39F7 Fab is shown as *white* surface representation. Selected KL1 residues and 39F7 Fab residues are shown in *sticks. E*, structure overlay of β-Klotho KL1 domain in complex with 39F7 Fab to full-length β-Klotho in complex with FGF21 C-terminal peptide (PDB ID:5VAQ). KL1 domain is shown as *blue* surface. The 39F7 Fab is shown as *green* cartoon for light chain and *purple* cartoon for heavy chain. FGF21 is shown as *red sphere* and KL2 domain is shown as *wheat* cartoon. *F*, structure overlay of β-Klotho KL1 domain in complex with 39F7 Fab to full-length α-Klotho in complex with FGF23 and FGFR1 (PDB ID:5W21). The KL1 domain is shown as *blue* surface. The 39F7 Fab is shown as *green* cartoon for light chain and *purple* cartoon for heavy chain. FGF23 is shown as *red sphere*. The KL2 domain is shown as *green* cartoon. FGFR1C is shown as *cyan* cartoon.

Antibody 39F7 interacts with KL1 extensively with the CDR loops from both heavy chain and light chain, including CDRL1, CDRL2, CDRL3, CDRH2, and CDRH3 (Fig. 4*A*). The epitope on the KL1 domain consists of residues from three surface loops, including loops α5/β6, α6/β7, and α7/β8, and residues from helices α4 and α7. The total buried solvent accessible area is 1177 Å^2^ of the Fab onto KL1 and 1320 Å^2^ of KL1 onto the Fab paratope. The Fab-KL1 interface involves both hydrophobic interactions and polar interactions. At the center of the interface, Trp-295 of KL1 sits in a hydrophobic pocket formed by the side chains of His-106, Tyr-107, Tyr-108, and Tyr-109 of CDRH3, and Tyr-33 of CDRL1 (Fig. 4*B*). In addition, 39F7 Fab makes a number of hydrogen bond (H-bond) interactions with six positively charged residues from KL1, including Arg-448 to Ser-56 side chain of CDRH2, Arg-411 to Tyr-33 hydroxyl of CDRL1, Arg-453 to Gly-93 backbone carbonyl of CDRL3, Tyr-265 to His-106 of CDRH3 at one site (Fig. 4*C*), as well as Arg-289 to Tyr-108 hydroxyl of CDRH3, Lys-293 to Ser-54 side chain of CDRL2, and Arg-364 to Thr-70 hydroxyl of LC framework at the second site (Fig. 4*D*). These extensive H-bond interactions provide the basis for the high-affinity binding to KL1.

Very recently, Lee *et al.* (17) reported the crystal structure of the full-length β-Klotho in complex with a FGF21 C-terminal peptide (PDB ID: 5VAQ). We compared the angle of approach of 39F7 Fab to the FGF21 C-terminal peptide in their recognition of the KL1 domain of β-Klotho. 39F7 Fab engages the KL1 domain from the opposite side of the FGF21 C-terminal peptide-binding site (Fig. 4*E*). Because the core domain of FGF21 is not present in the complex structure of β-Klotho with FGF21 C-terminal peptide (17), we performed comparative analysis with the complex structure of α-Klotho with FGF23 and FGFR1c (PDB ID: 5W21) (16) to assess the FGF21 core domain binding. As shown in Fig. 4*F*, superposition of the two structures shows that the core domain of FGF23 is positioned in close proximity to the KL2 domain with no close interactions with the KL1 domain. Apparently, 39F7 competes with neither FGF ligand nor receptor FGFR1c, in consistency with our *in vitro* analysis.

### EM studies of β-Klotho alone or complexed with FGF21 or 39F7 Fab

**Figure 5.**
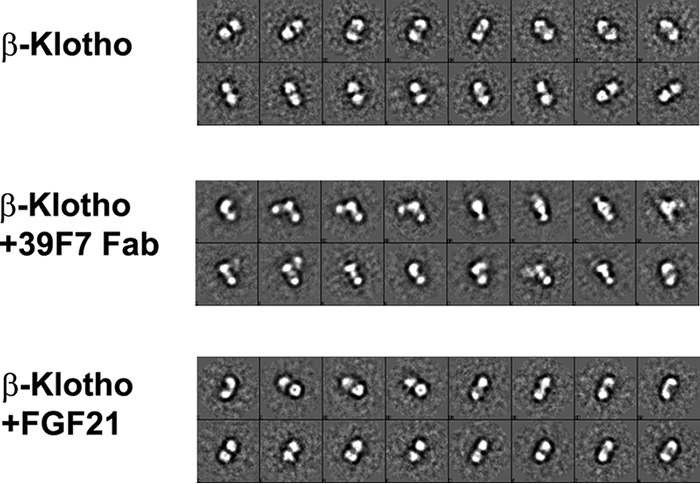
**EM characterization of β-Klotho and its interactions with 39F7 antibody and ligand FGF21 in solution.** 2D class averages are shown for full-length β-Klotho (*top panel*, labeled *KL*), β-Klotho + 39F7 Fab (*middle panel*, labeled *KL* + *Fab*), and β-Klotho + FGF21 (*bottom panel*, labeled *KL* + *FGF21*).

Despite the extensive efforts to crystallize the full-length β-Klotho protein, we were not able to obtain diffraction-quality crystals. We utilized negative stain EM to probe the overall conformation of the full-length β-Klotho protein and its complex with ligand or antibody in solution. Inspection of the raw EM images suggests that the full-length β-Klotho shows a highly monodispersed population. Image classification and 2D class average reveal a majority of full-length β-Klotho molecules as two equal-sized blobs in an extended arrangement (Fig. 5). Although the structure flexibility between the two domains prevents 3D reconstruction, two important findings were evident from the 2D class average. First, the full-length β-Klotho extracellular domain (ECD) exists in monomeric form. In the absence of ligand or receptor, there is no higher order of molecular species. This agrees with the size-exclusion chromatography profile of full-length β-Klotho demonstrating a monomeric form (Fig. S2). Second, the KL1 and KL2 domains adopt an extended conformation. The domain arrangement shows that one domain sits on top of the other with limited conformational flexibility, supporting the recently published structures of β-Klotho and α-Klotho (16, 17).

Next, we examined the binding of natural ligand and antibody to β-Klotho using negative stain EM. The 2D average of 39F7 Fab and β-Klotho complex shows three distinctive blobs of similar size (Fig. 5). The molecular mass of the Fab, KL1, and KL2 domain are all about 50 kDa, making it difficult to distinguish the position of KL2 and Fab relative to the KL1 domain. However, the EM micrographs show that 39F7 Fab approaches β-Klotho in a perpendicular angle to the KL1-KL2 tandem arrangement (Fig. 5). Thus, the arrangement of 39F7 to full-length β-Klotho in solution indeed agrees with what was observed from crystal structural comparison (Fig. 4*E*).

The EM micrographs of FGF21 and β-Klotho complex mixture, however, show predominantly only a two-lobe structure in extended conformation which is very similar to that of the apoβ-Klotho. The lack of FGF21 feature in the EM micrograph is probably because of the small size of FGF21 and the low resolution of the negative stain EM. On the other hand, it is obvious that FGF21 neither induces significant conformational changes to β-Klotho nor promotes higher order complex formation. Again, this solution state data of FGF21 with β-Klotho is consistent with the crystal structure observation in which the key recognition of FGF21 to β-Klotho KL1 is through its C-terminal peptide (17). The core domain of FGF21 seems to have no direct interactions with β-Klotho and is likely flexible in the solution, resulting in no density in the EM. Taken together, it is evident that β-Klotho protein adopts a stable tertiary structure in the presence of both natural agonist FGF21 and agonistic antibody such as 39F7 in solution.

## Discussion

The endocrines FGF19, FGF21, and FGF23, have great potential to be important therapeutics for a variety of human diseases such as obesity, type II diabetes, and kidney and cardiovascular diseases (4). FGF21, in particular, has shown efficacy in improving insulin action, glycemic control, inducing plasma lipid reduction, and inducing weight loss in humans (21, 25). For a long time, the lack of structural information of FGF21, β-Klotho, or the complex of FGF21/β-Klotho/FGFR impeded our understanding of this signaling pathway. The recently published structures of endocrine FGFs with Klotho proteins provide molecular insights into how a natural agonistic ligand such as FGF21 recognizes the co-receptors β-Klotho and how FGF23 recognizes both FGFR1c and α-Klotho (16, 17). These structures also facilitate a better understanding of the mechanism of the agonistic antibody in this report in the context of the natural agonist.

In the current study, we identify 39F7, an agonistic antibody that is able to induce receptor signaling *in vitro* and demonstrate the molecular mechanism underlying its agonistic nature, resembling FGF21 function. 39F7 activates β-Klotho/FGFR1c receptor complex specifically, but not the other FGFRs, even though direct interaction between 39F7 and FGFR1c was not detected. The exact mechanism of the receptor specificity is not clear. It is possible that 39F7 recognizes a β-Klotho conformation that favors FGFR1c binding, or that a weak interaction between 39F7 and FGFR1c can be augmented by β-Klotho.

The crystal structure of KL1 in complex with 39F7 Fab not only reveals an agonistic mAb binding to KL1, but also the structural architecture of KL1 itself. Although the overall fold of KL1 resembles closely that of KLrP, the architecture of the active site of KL1 deviates from that of KLrP, confirming that KL1 is unlikely an enzymatic protein with glycosidase activity (26). Instead, KL1 serves as a scaffold partner for the complex of ligand FGF21 and its receptor FGFR1c for downstream signaling. This is in agreement with the recent published structures (16, 17) where it is shown that the putative substrate-binding site in Klotho is mutated to be a binding site for FGF21 C terminus.

**Figure 6.**
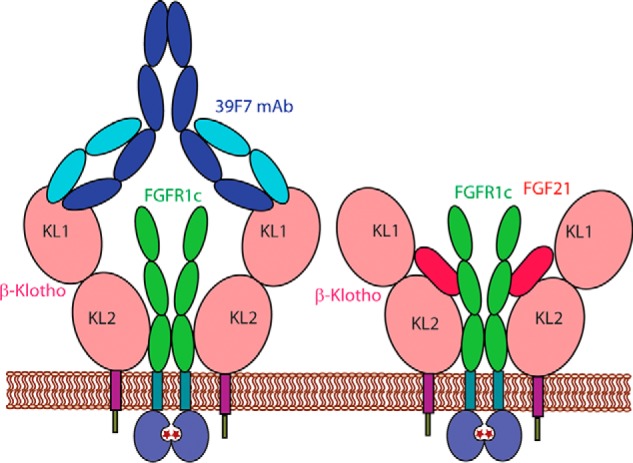
**Model of the mechanism of action for 39F7 antibody activation of β-Klotho and FGFR1c.**

Our biochemical analysis demonstrates that bivalent 39F7 IgG but not the monovalent 39F7 Fab can activate the receptor signaling. Such a bivalency requirement from the 39F7 antibody suggests that 39F7 IgG binding to β-Klotho promotes the formation of a physiologically active receptor dimer complex. The active receptor complex induced by 39F7, however, might be different from the receptor complex induced by FGF21 because 39F7 and FGF21 utilize different surfaces on β-Klotho. For paracrine FGFs, FGFR dimerization is required for signaling (27) and the dimerization of FGFR is greatly enhanced by the co-factor heparin. Less is known on the endocrine FGF family members, particularly because β-Klotho is a 100 kDa protein and would be incompatible with the heparin-binding site in the FGF/FGFR/heparin ternary complex. Examination of the published full IgG crystal structures revealed that the two Fab fragments in an intact IgG are separated by a distance of ∼120–150 Å (PDB IDs: 1HZH, 1IGT, 1IGY). Our EM studies suggest that the 39F7 Fab fragment approaches full-length β-Klotho in a perpendicular angle. Together with the data that the KL1 and KL2 domains are arranged in an extended conformation, we propose a model of 39F7 mAb/β-Klotho dimer in which the two β-Klotho molecules are tethered together (Fig. 6). The distance between the two β-Klotho molecules is about 100 Å. An FGFR1c/FGF dimeric complex has a dimension of 80–100 Å and would fit between the two β-Klotho molecules. In this model, the binding of the agonistic mAb 39F7 to β-Klotho prompts the dimerization of FGFR1 and each β-Klotho molecule might potentially interact with one or more domains of D1, D2, and D3 of FGFR for downstream signaling, resembling the arrangement of the ternary complex formed by FGF23, α-Klotho, and FGFR1 (16). Although alternative models of the ternary complex are possible, this model provides a molecular basis to understand the agonistic antibodies against β-Klotho mimicking FGF21 activities.

Finally, the nature of 39F7 recognition of β-Klotho through a FGF21-independent manner may present an attractive opportunity for this agonistic antibody as a therapeutic for the treatment of various cardiometabolic diseases, in a different mechanism from FGF21 or FGF21 competitive therapeutic molecules. Compared with protein-based therapeutics such as engineered FGF21, mAb 39F7 offers unique advantages of antibody therapy such as longer half-life, low dosing requirement, and high specificity that avoids off-target liability. Moreover, an antibody targeting co-receptor β-Klotho might avoid the potential on-target side effects of the engineered natural ligand FGF21 because of the complex biological network in which endocrines partake. Although mAb 39F7 could prove beneficial, further studies are needed to fully evaluate its potential use as a therapeutic for treating various metabolic diseases such as diabetes.

## Materials and methods

### Solution equilibrium-binding assay

Binding affinity of 39F7 to human β-Klotho was characterized by solution equilibrium-binding assay on a Biacore T200 instrument. Briefly, 1 nm or 3 nm human β-Klotho were incubated with a series concentration of 39F7 (ranging from 0.007 to 62.5 nm) in a PBS solution containing 0.1 mg/ml BSA and 0.005% Surfactant P20 for 4 h at room temperature. Binding of the free β-Klotho in the mixed solutions was measured by injecting the solutions over the amine immobilized 39F7 surface. Binding of 1 nm or 3 nm β-Klotho alone was used as 100% binding, and the relative binding of β-Klotho in the mix with 39F7 was calculated. Plotting the free β-Klotho–binding signal *versus* antibody concentration, the *K_D_* was obtained from nonlinear regression of the competition curves using an *N*-curve one-site homogeneous binding model in KinExA Pro software. The detailed mathematical equation and numerical simulation were described by Glass and Winzor (28). Previous studies have shown that in equilibrium-binding assay, valence of the binding protein had no influence on the value of *K_D_* (29).

### Kinetic-binding assay

Binding of 39F7 and FGF21 to human β-Klotho and FGFR1c was characterized by kinetic-binding assay on a Biacore T200 instrument. Briefly, a mouse anti-His antibody was immobilized on a CM5 chip by amine coupling to around 6000 RU. His-tagged human β-Klotho and FGFR1c were captured to an approximate density of 40 RU and 60 RU on the anti-His surface. 39F7 (100 nm) and FGF21 (500 nm) diluted in a PBS solution containing 0.1 mg/ml BSA and 0.005% Surfactant P20 were injected over the captured β-Klotho and FGFR1c to measure their binding. The sensor gram was double referenced by subtracting blank surface and sample buffer.

### Competition-binding assay

The competition-binding activity of 39F7 to FGF21 on the binding to human β-Klotho was characterized by measuring the binding of free β-Klotho to the immobilized FGF21 surface on a Biacore T200 instrument. Briefly, 10 nm human β-Klotho was incubated with 10 nm and 100 nm 39F7 or FGF21 in a PBS solution containing 0.1 mg/ml BSA and 0.005% Surfactant P20 for 1 h at room temperature. Binding of the free β-Klotho in the mixed solutions was measured by injecting the solutions over the amine immobilized FGF21 surface. Binding of 10 nm β-Klotho alone was used as 100% binding, and the relative binding of β-Klotho in the mixture with 39F7 or FGF21 was calculated.

For solution equilibrium-binding assay and competition-binding assays, surfaces were regenerated by injection of 10 mm glycine, pH 2.0, at 30 μl/min for 30 s. For kinetic-binding assay, surfaces were regenerated by injection of 10 mm glycine, pH 1.5, at 30 μl/min for 30 s.

### In vitro FGF-signaling assays

The CHO reporter cell assay and the L6 pERK-signaling assay were performed as described previously (30).

### Protein expression

β-Klotho KL1 construct (residues 52–521) was cloned to pFastBac plasmid and the virus was generated. TNO cells (Orbigen) were maintained in 2.8-liter Fernbach flasks at densities 0.5 to 4 × 10^6^ cells/ml shaking at 125 rpm at 27 °C. Cells were transferred to a 22-liter WAVE bag (GE Healthcare) at a density of 0.8 × 10^6^ cells/ml in a volume of 10 liters ESF921 culture media (Expression Systems LLC). The cell bag was placed on the WAVE bioreactor rocker and shaken at 9°, 25 rpm at 27 °C. After 24 h the cells had reached a density of 2 × 10^6^ cells/ml +90% viability and were infected with a virus stock at a multiplicity of infection of 0.05. Kifunensine (Biomol GmbH) was added to 5 μm. Culture was harvested after 72 h and cells pelleted at 3000 × *g* for 10 min on a Sorvall centrifuge. The supernatant was clarified on a 1 μm serum filter (Pall Corp.) then bound to a 5 ml Ni Sepharose excel column at 5 ml/min using the sample pump on an AKTA purifier (GE Healthcare). The column was washed for 80 column volumes with a buffer of 50 mm Tris, pH 7.9, 250 mm NaCl, and 10% glycerol. Recombinant KL1 was eluted over a linear gradient of 0–250 mm imidazole in a buffer of 50 mm Tris, pH 7.9, 250 mm NaCl, and 10% glycerol.

### Protein purification and crystallization

The protein was further purified by anion exchange column (Source Q; GE Healthcare) using a 0–1 m NaCl gradient in a buffer containing 50 mm Tris, pH 7.4, 10% glycerol, followed by cleavage with Endo H enzyme (New England Biolabs) to remove glycosylation. The protein was then put on a cation exchange column (MonoS; GE Healthcare) and eluted with 0.02–1 m NaCl gradient in buffer containing 50 mm Hepes, pH 6.8, and 10% glycerol. The purified protein was complexed with the 39F7 Fab in a molar ratio of 2 Fab molecules to 1 β-Klotho KL1 molecule and purified by size-exclusion chromatography (Superdex 200; GE Healthcare) in buffer containing 25 mm Hepes, pH 6.8, 150 mm NaCl, and 10% glycerol. The purified complex was then concentrated to 8 mg/ml for crystallization. The β-Klotho/Fab complex was crystallized by sitting drop vapor diffusion method at 20 °C with 1:1 protein solution to reservoir solution of 20% PEG 3350 and 0.2 m sodium sulfate. The crystals were transferred into the mother liquor with stepwise cryo-protection of 10, 20, and 30% ethylene glycol and were flash frozen with liquid nitrogen.

### Structure solution

The X-ray diffraction data sets were collected at the synchrotron beamline 502 at Advanced Light Source (ALS) in Berkeley, California. The data were integrated using XDS and scaled using Aimless (31) in the CCP4 program suite. The structure was solved by molecular replacement with PHASER (32) using a previously solved internal structure of the 39F7 Fab and an internally produced model of KL1 as search models. Model building was carried out in Coot (33) and refinement was done in REFMAC5 (34) in the CCP4 program suite (35). All structural figures were prepared using Pymol. The data collection and refinement statistics are presented in Table 1.

**Table 1:** **Data collection and refinement statistics**

	KL1–39F7 Fab complex*^a^*
**Data collection**	
Space group	P 21
Unit cell	118.02 68.47 147.81 90 111.87 90
Wavelength (Å)	1.000
Resolution range (Å)	19.8–2.7 (2.79–2.7)
No. of total reflections	119,874 (11,884)
No. of unique reflections	60,303 (5986)
Multiplicity	2.0 (2.0)
Completeness (%)	99 (99)
Mean *I*/σ(*I*)	7.58 (1.56)
Wilson *B*-factor (Å^2^)	44.54
*R*_sym_	0.082 (0.4659)

**Refinement**	
No. of reflections	60,283 (5982)
*R*_work_/*R*_free_	0.1838/0.2672
Number of nonhydrogen atoms	13,563
Macromolecules	13,279
Ligands	124
Average *B*-factor (Å^2^)	50.14
Protein	50.28
Solvent	32.40
r.m.s.d in bond length (Å)	0.014
r.m.s.d in bond angles (°)	1.33

*^a^* Values in parentheses are for the highest resolution shell.

### EM

For negative stain imaging, purified β-Klotho or β-Klotho/Fab 39F7 complex was diluted to 10 μg/ml in 25 mm Hepes, pH 7.5, and 150 mm NaCl. Five molar ratio of FGF21 was added to 10 μg/ml β-Klotho to make FGF21/β-Klotho complex. Continuous carbon grids (CF300-Cu-UL, Electron Microscopy Sciences) were plasma cleaned for 30 s. Then, 4 μl of sample was applied and wicked away before staining with 2% uranyl acetate (36). Images were manually acquired on an FEI Talos F200C operated at 200 kV, at a nominal magnification of 57,000× on a Ceta camera with a pixel size of 2.58 Å/pixel. All image processing was carried out with the EMAN2 package (37). Particles were picked with e2boxer and CTF correction was carried out on a per particle basis with e2ctf. The CTF corrected particles were then subjected to reference-free 2D classification using e2refine2d.

## Author contributions

X. M., Y. L., and Z. W. conceptualization; X. M. data curation; X. M., J. W., S. J., W. W., X. Y., Y. L., and Z. W. formal analysis; X. M., Y. L., and Z. W. supervision; X. M., J. W., S. J., W. W., X. Y., W. R., S. T., Y. L., and Z. W. investigation; X. M. methodology; X. M., Y. L., and Z. W. writing-original draft; X. M., Y. L., and Z. W. writing-review and editing.

## Supplementary Material

Supporting Information

## Footnotes

4 The abbreviations used are:

ECM: extracellular matrix
HS: heparan sulfate
FGFR: FGF receptor
mAb: monoclonal antibody
SPR: surface plasmon resonance
r.m.s.d.: root mean square deviation
KLrP: KL-related protein
CDRs: complementarity determining regions
H-bond: hydrogen bond
RU: response unit
pERK: phosphorylated ERK.
