# Supplementary material for: Agonistic β-Klotho antibody mimics fibroblast growth factor 21 (FGF21) functions
Source: J Biol Chem. 2018 Aug 1;293(38):14678–88. doi: 10.1074/jbc.RA118.004343 (PMC6153294; doi:10.1074/jbc.RA118.004343)
Supplement: Supporting Information [file supp_293_38_14678__index.html]

Agonistic β-Klotho antibody mimics fibroblast growth factor 21 (FGF21) functions — Agonistic antibody binding to β-Klotho — Agonistic β-Klotho antibody mimics fibroblast growth factor 21 (FGF21) functions — Agonistic antibody binding to β-Klotho — Supporting Information 

# Agonistic β-Klotho antibody mimics fibroblast growth factor 21 (FGF21) functions

## Supporting Information

- Supporting Information - Supporting Information
