# Supplementary material for: Agonistic β-Klotho antibody mimics fibroblast growth factor 21 (FGF21) functions
Source: J Biol Chem. 2018 Aug 1;293(38):14678–88. doi: 10.1074/jbc.RA118.004343 (PMC6153294; doi:10.1074/jbc.RA118.004343)

Figure S1: Structure superposition of KL1 domain. KL1 domain in complex with 39F7 Fab is colored in blue and KL1 domain in PDB 5VAK is colored in pink.

Figure S2. SEC chromatogram of full length β-Klotho on Superdex G200 10/300.

Figure S1


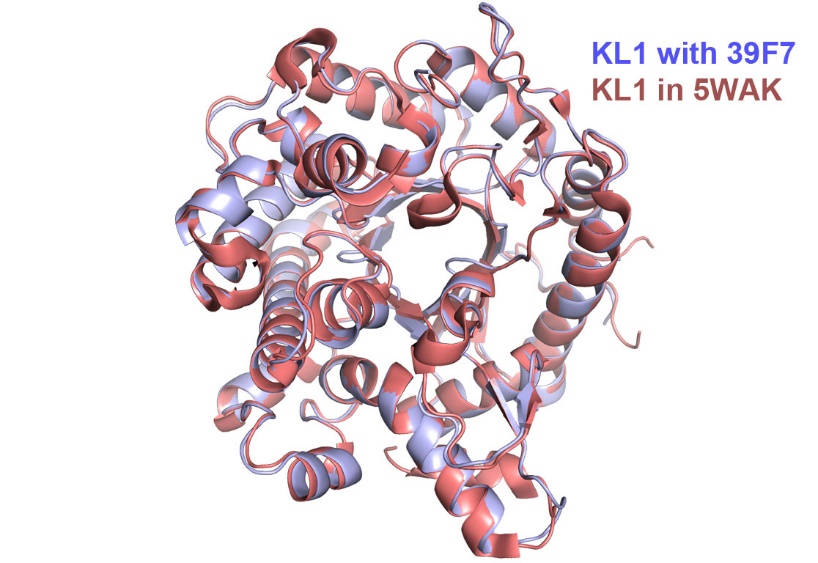


Figure S2


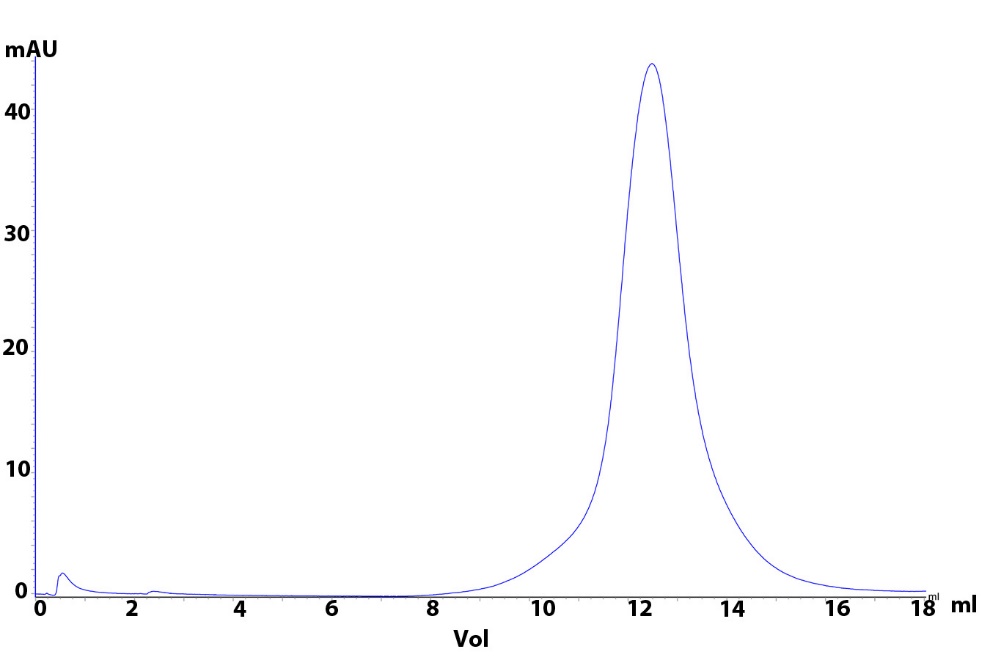

Supplement: Supporting Information [file supp_RA118.004343_138667_2_supp_175726_pcjckx.docx]
